# Supplementary material for: Trace element systematics constrain the origin of fluids that form gem-quality diamonds
Source: Commun Earth Environ. 2026 May 29;7(1):469. doi: 10.1038/s43247-026-03662-1 (PMC13221304; doi:10.1038/s43247-026-03662-1)
Supplement: Supplementary file 2 — Supplementary Information [file 43247_2026_3662_MOESM2_ESM.pdf]

Trace element systematics constrain the origin of fluids that form gem-quality diamonds

Aleksandr Rakipov[1,2,3*], Alan B. Woodland[1,2], Fabrizio Nestola[3], Matilde Galie[1,3], Martha G. Pamato[3], Davide Novella[3], Maxwell C. Day[3], Tobias Erhardt [1,2], Wolfgang Müller[1,2]

[1]*Institut für Geowissenschaften, Goethe University, 60438 Frankfurt am Main, Germany*

[2]*Frankfurt Isotope and Element Research Center (FIERCE), Goethe University, 60438 Frankfurt am Main, Germany*

[3]*Department of Geoscience, University of Padova, 35131 Padova, Italy*

Corresponding author: Aleksandr Rakipov; e-mail: rakipov@fierce.uni-frankfurt.de

Supplementary Information

The following document provides Supplementary Figures 1-10 showing diamond samples, Raman maps, and depth profiles filtered for the SFR signal, as well as Supplementary Figures of all LA-ICPMS depth profiles referenced in the main article.

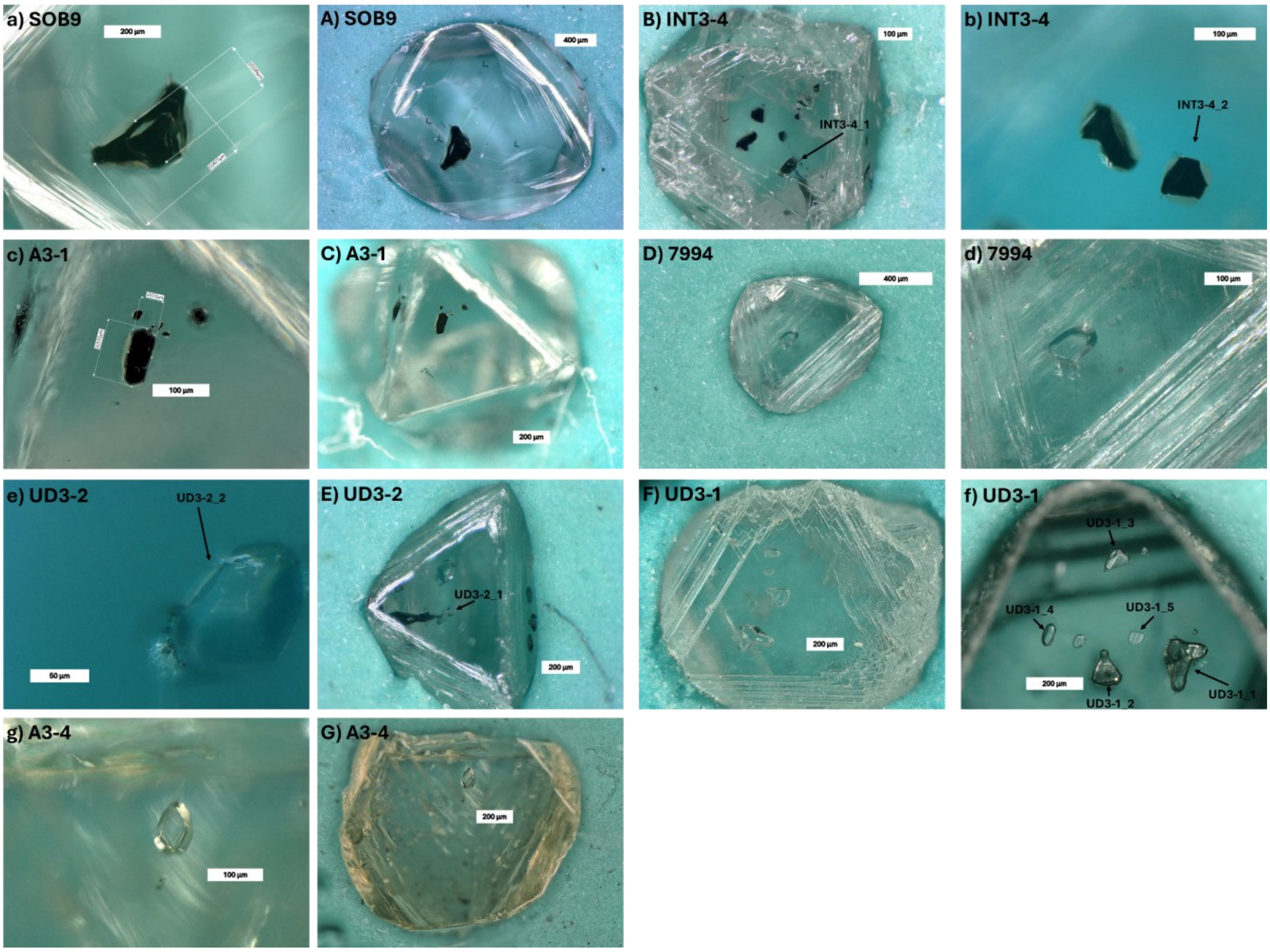

Supplementary Figure 1 Diamond samples and their mineral inclusions. *Capital letters (A-G) show whole-sample images, while small letters (a-g) present enlarged views of inclusions from the corresponding host diamond.*

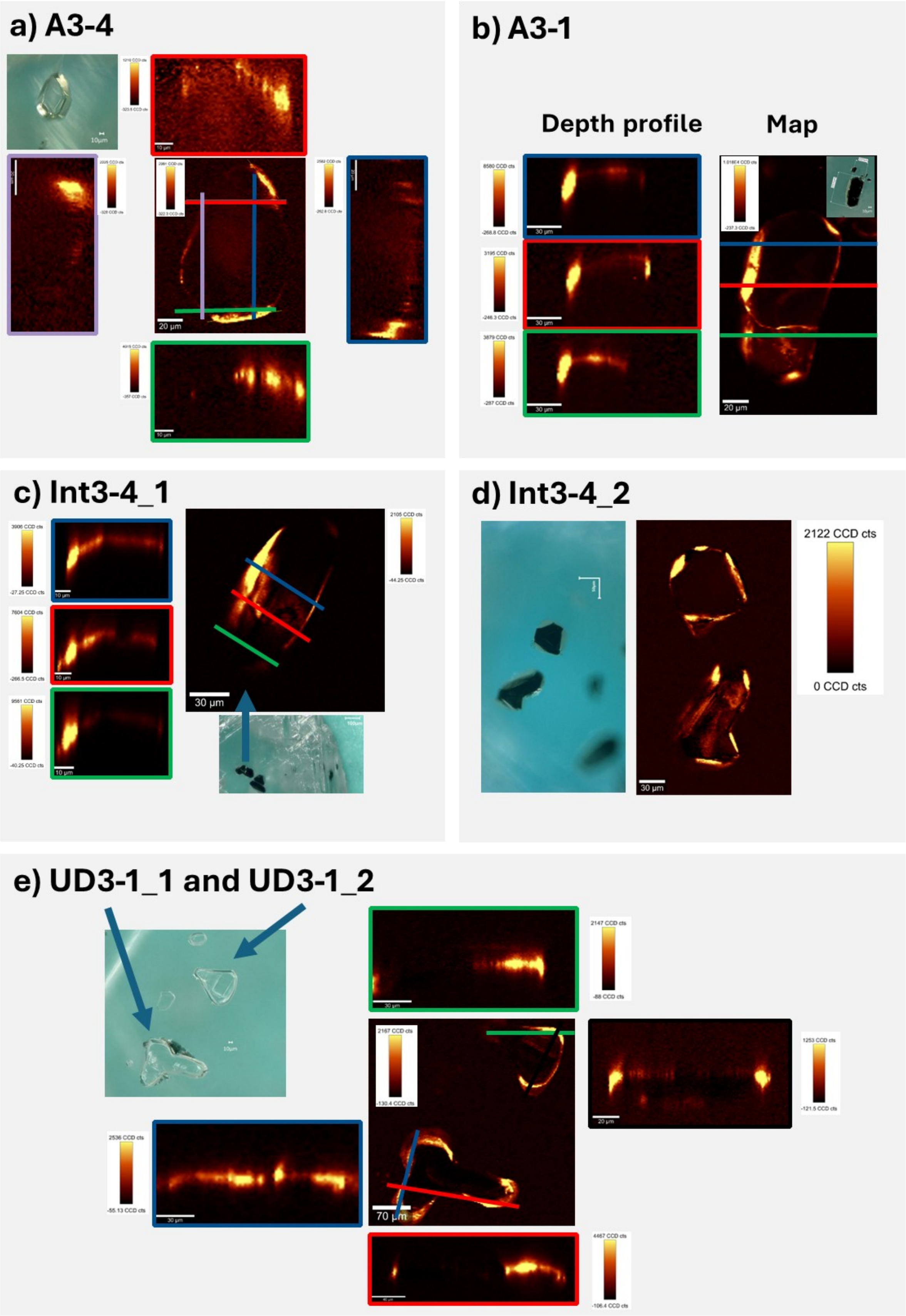

Supplementary Figure 2 SFR distribution around inclusions A3-4, A3-1, INT3-4_1, INT3-4_2, UD3-1_1, and UD3-1_2. *Raman map filtered for the indicative SFR signal, shown together with colour-coded depth profiles across omphacitic clinopyroxene in (a), magnesiochromite (b-d), and olivine (e) inclusions. The SFR distribution is classified as enveloping for inclusions in (b) and (c) and patchy in (a) and (e). See Figure 1 for mineral inclusion and SFR spectra.*

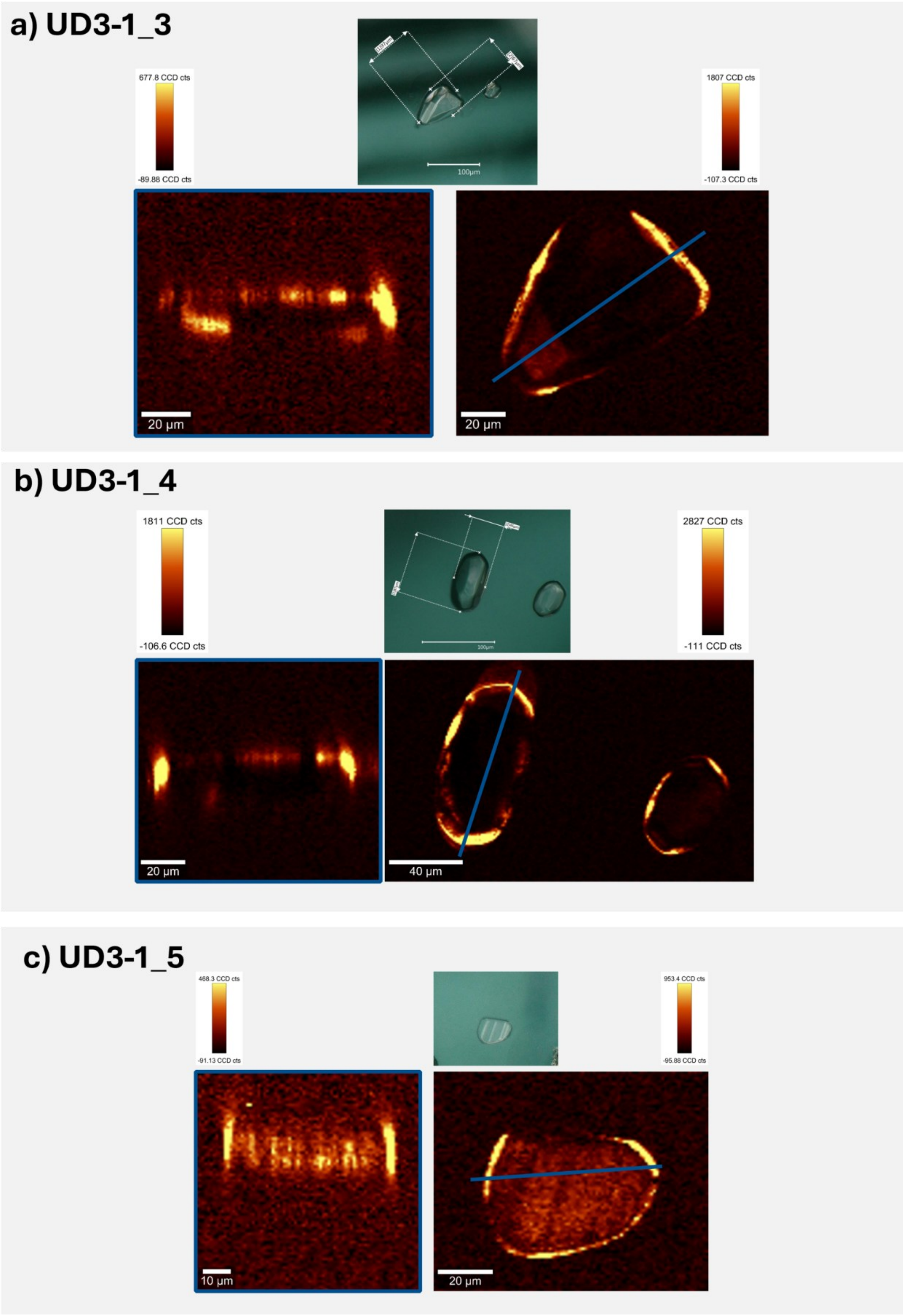

Supplementary Figure 3 SFR distribution around olivine inclusions UD3-1_3, UD3-1_4, and UD3-1_5. *Raman map filtered for the indicative SFR signal, with depth profiles indicated in blue. The fluid distribution is classified as enveloping for all inclusions. See Figure 1 for mineral inclusion and SFR spectra.*

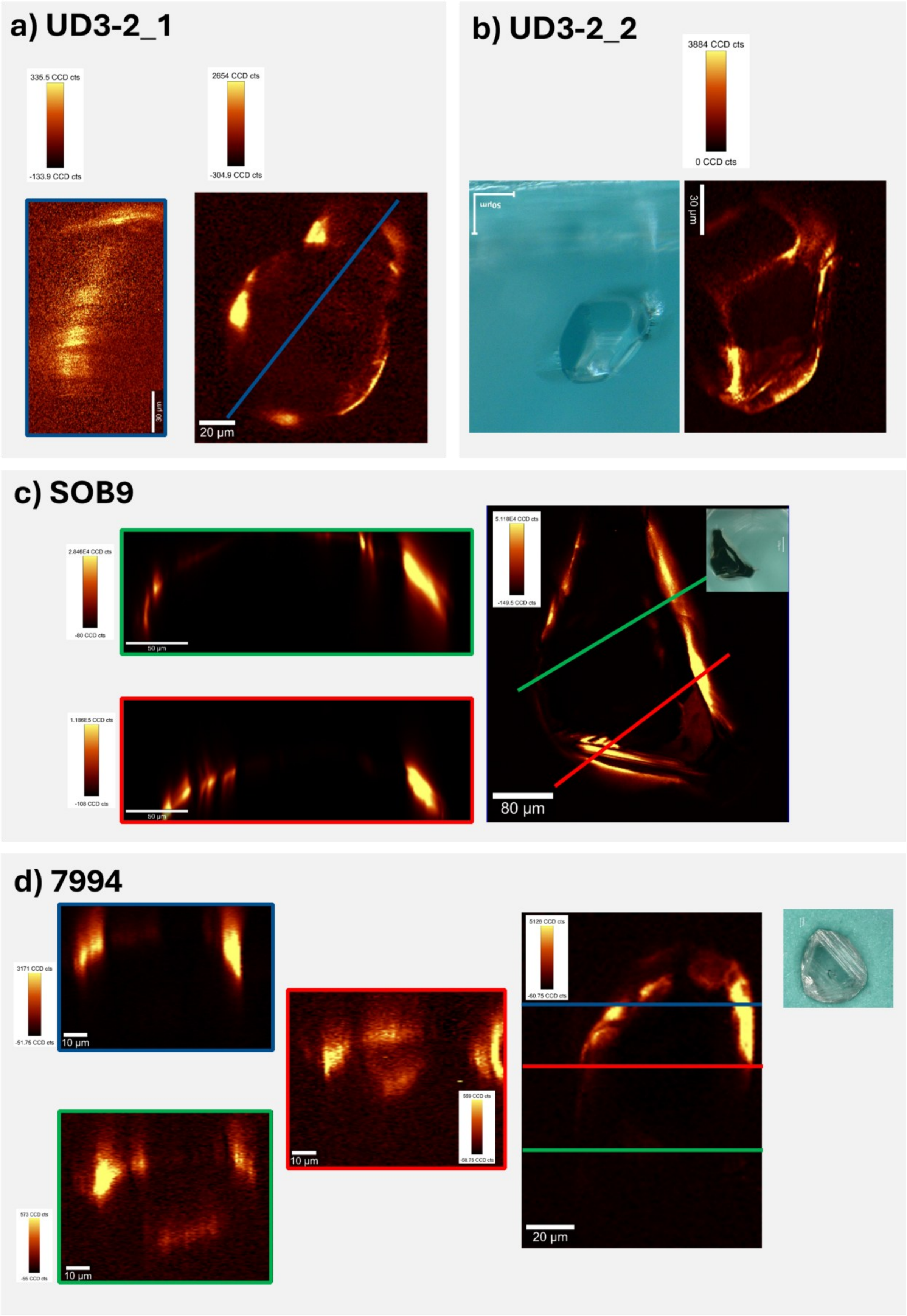

Supplementary Figure 4 SFR distribution around inclusions UD3-2_1, UD3-2_2, SOB9, and 7994. *Raman map filtered for the indicative SFR signal, with different depth profiles according to colour across olivine in (a-b), (d), and magnesiochromite inclusions in (c). The distribution is classified as enveloping for inclusions in (a) and (d) and as patchy in (c). See Figure 1 for mineral and SFR inclusion spectra.*

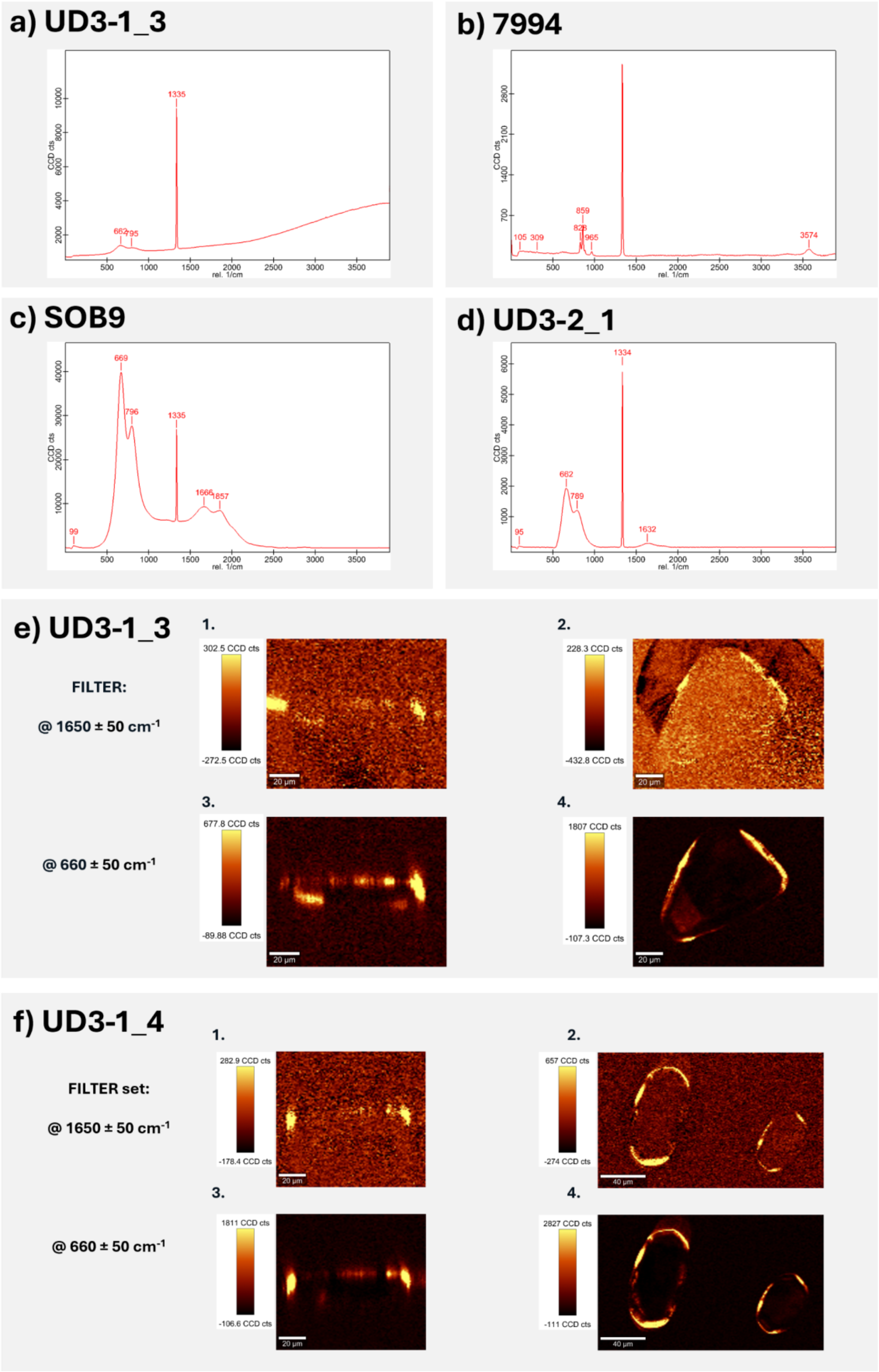

Supplementary Figure 5 Spectral evidence for the hydrous component: *(a) broad absorption previously attributed to OH-stretching by Nimis et al.*[1]*; (b) OH-stretching peak at 3574 cm^{-1} due to olivine of sample 7994*[2]*; (c, d) SFR signal and broad peaks at ~1650 cm^{-1} that may be due to H_2O-bending for inclusions SOB9 and UD3-2_1*[3]*. (e, f) Raman maps filtered for the ~1650 cm^{-1} band (H_2O-bending; in panels 1. and 2.) and the ~660 cm^{-1} band (SFR; in panels 3. and 4.).*

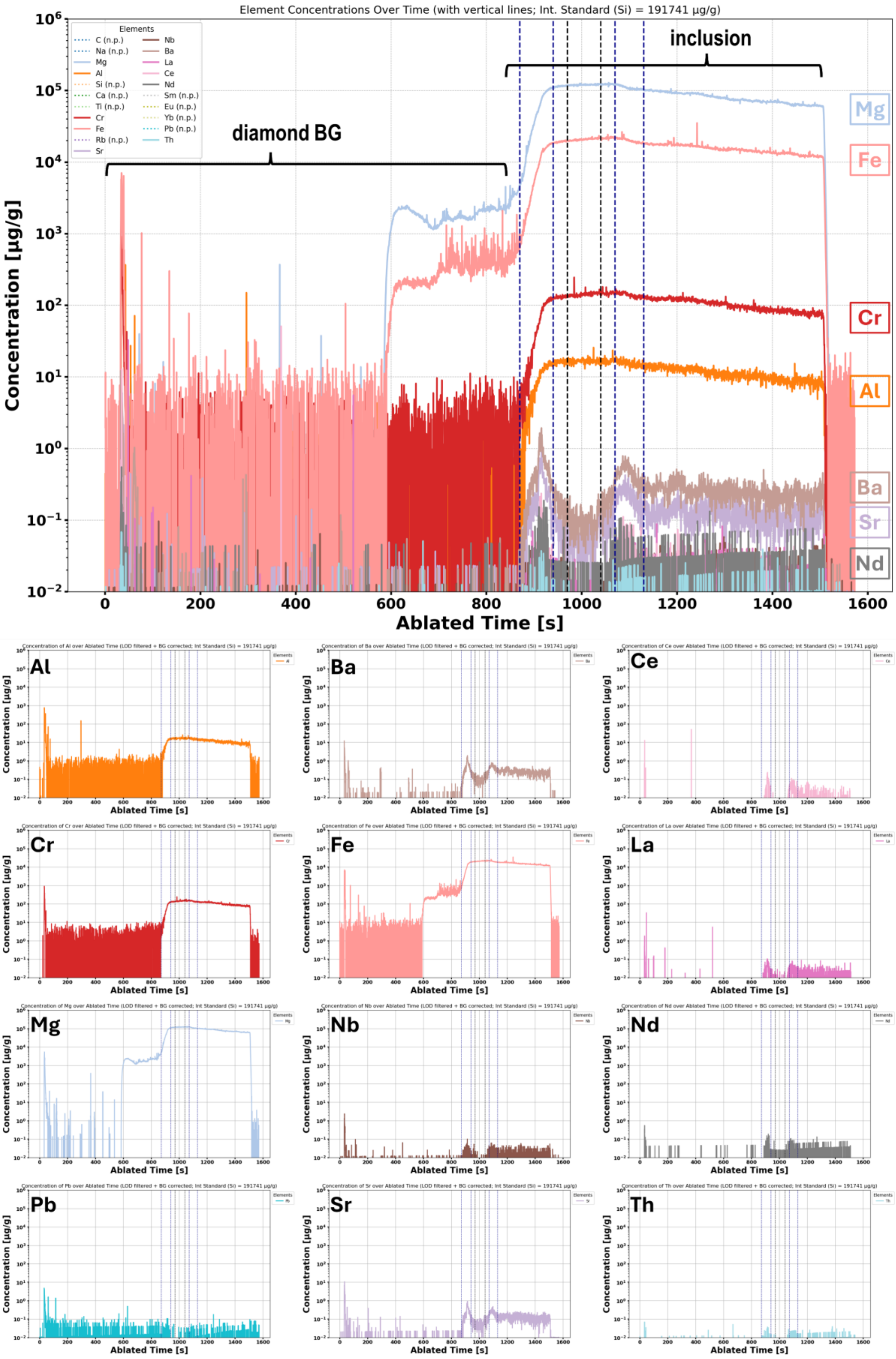

Supplementary Figure 6 Chemical depth profile of olivine inclusion (7994). *The upper panel shows the multi-element depth profile of the inclusion 7994, while the panels below display individual single-element depth profiles*

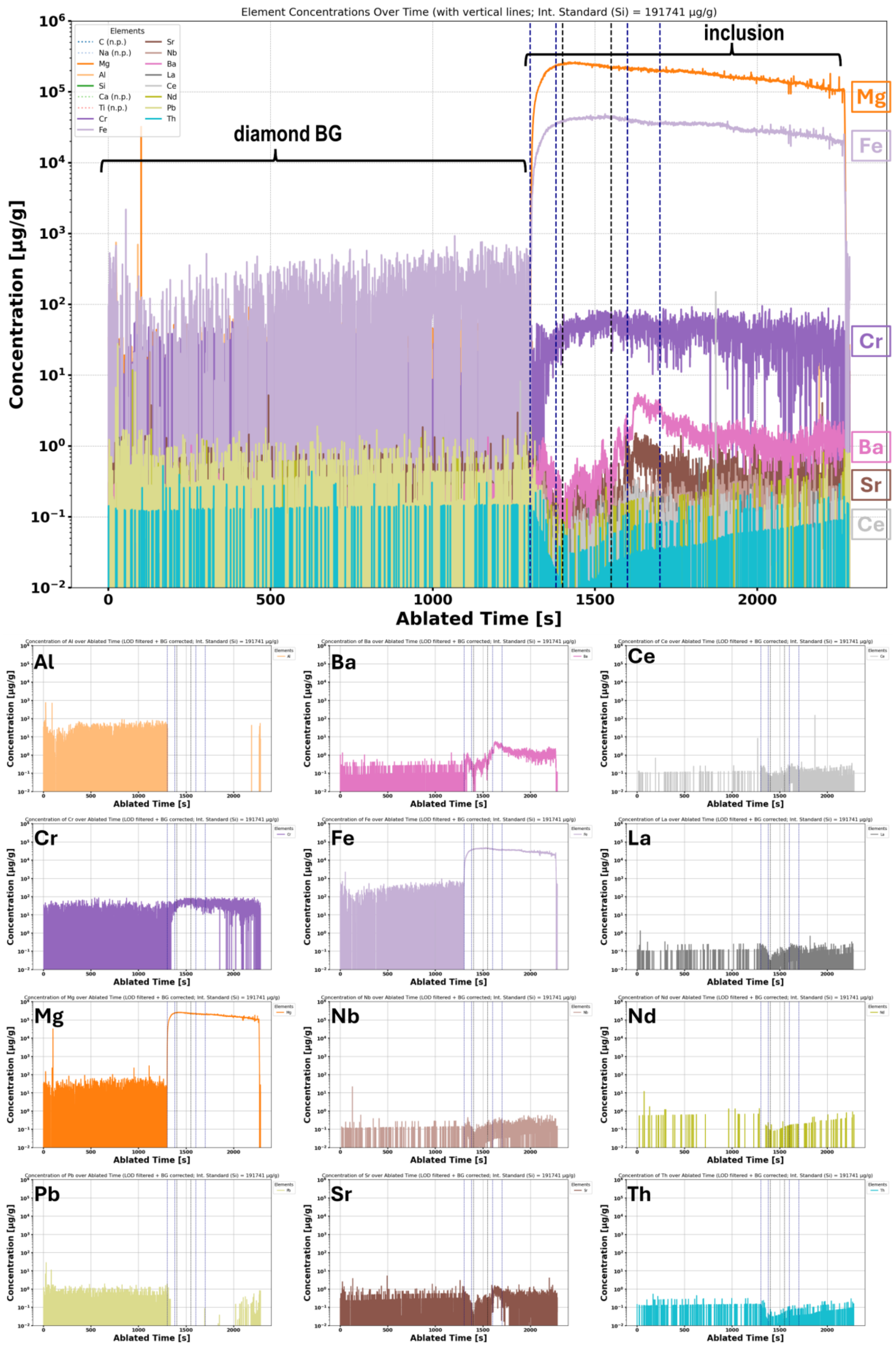

Supplementary Figure 7 Chemical depth profile of olivine inclusion (UD3-1_4). *The upper panel shows the multi-element depth profile of the inclusion UD3-1_4, while the panels below display individual single-element depth profiles.*

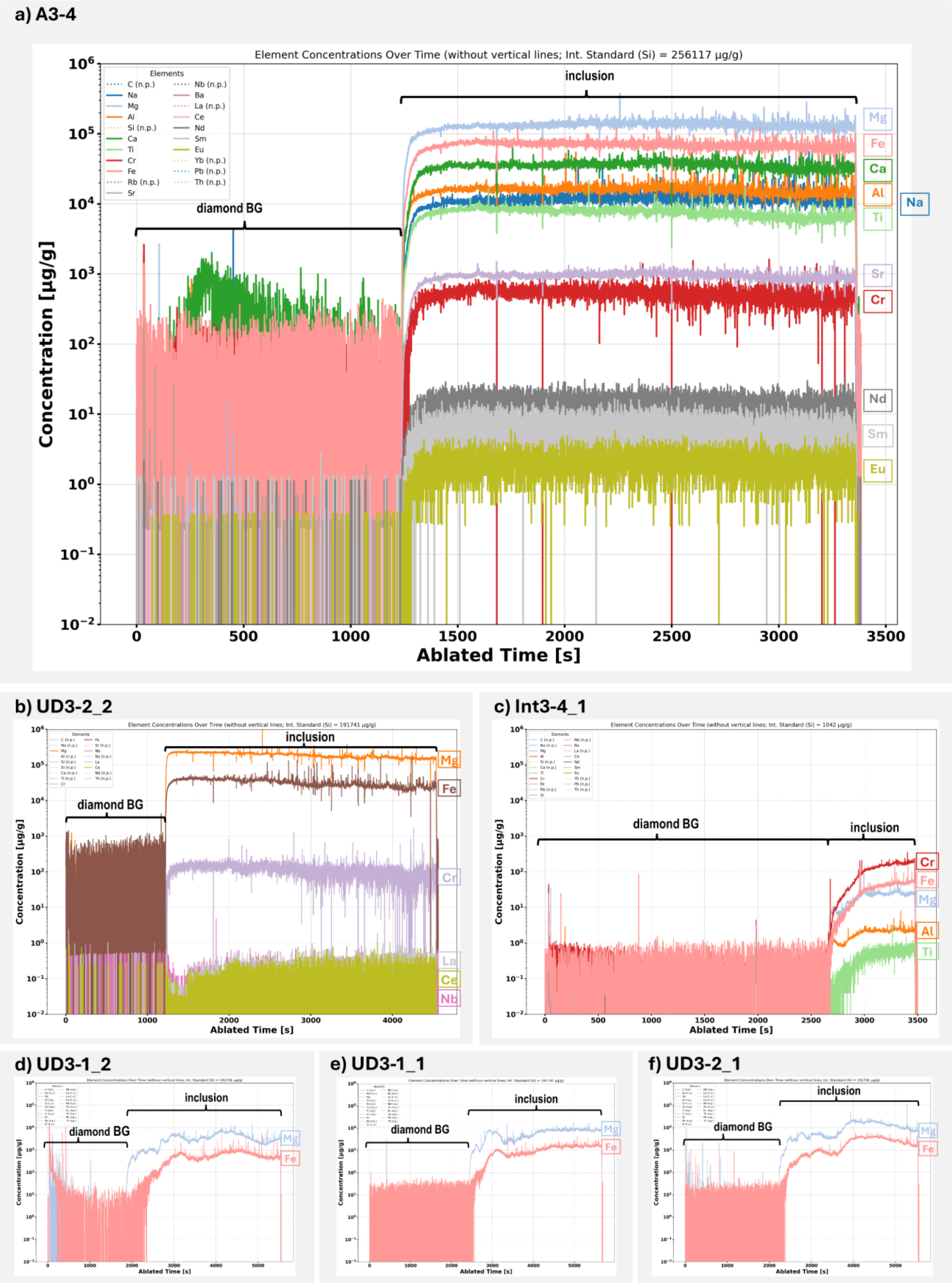

Supplementary Figure 8 Chemical depth profiles of inclusions A3-4, UD3-2_2, Int3-4_1, UD3-1_2, UD3-1_1, and UD3-2_1. *Omphacitic clinopyroxene (a) and the olivine inclusion (b) show group 2 depth profiles, in which the mineral inclusion and the SFR cannot be resolved, likely due to a patchy SFR distribution or the high trace-element background of the omphacitic clinopyroxene. The magnesiochromite inclusion (c) and the olivine inclusions in (d-f) display group 1 depth profiles, in which only major-element (Mg, Al, Ti, Cr, and Fe) signals were detected, and do not allow separation of the mineral inclusion from the SFR.*

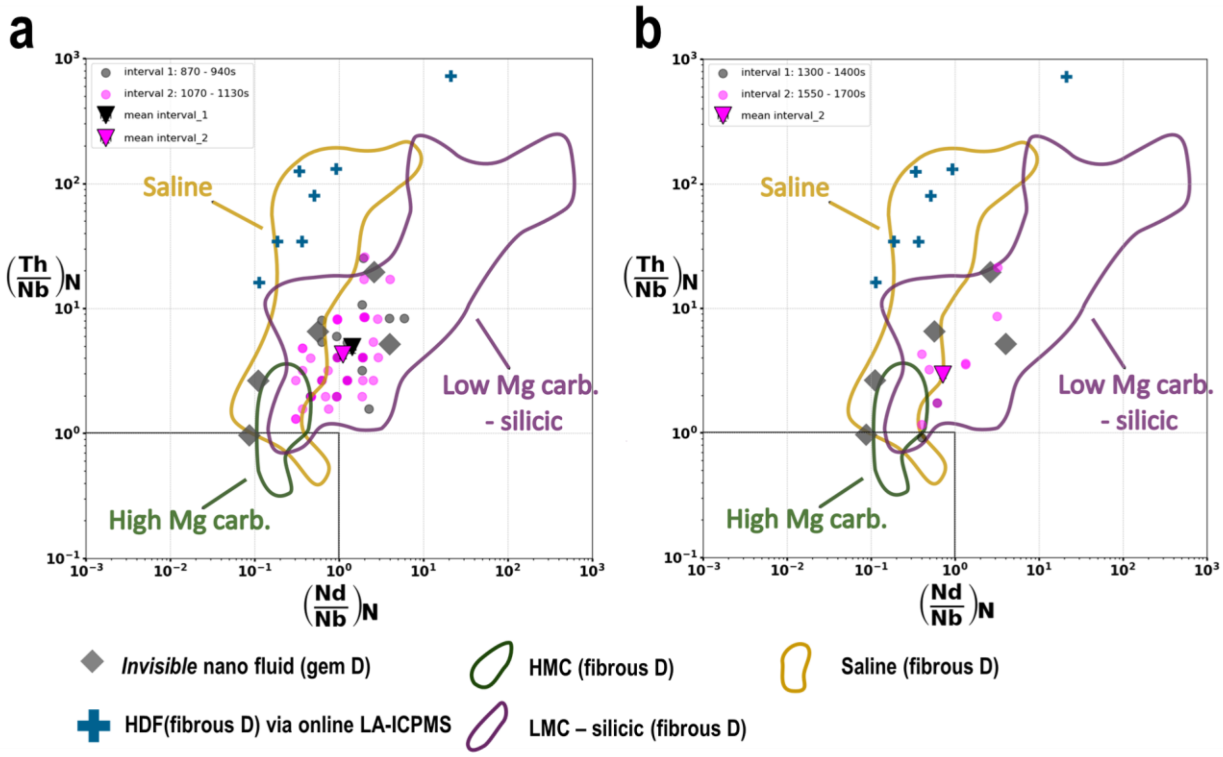

Supplementary Figure 9 Primitive mantle-normalised data extracted from the SFR-related signal of sample 7994 (a) and UD3-1_4 (b), shown in $(Nd/Nb)_N$_$(Th/Nb)_N$ space. *Black and magenta dots represent data from the first (above olivine inclusion) and second (below-inclusion) fluid intervals, corresponding to the black and magenta dotted lines in the depth profiles (Fig.1), triangles indicate the means. The standard error (SE=σ/sqrt(n)) is represented by error bars; if not visible, the error is smaller than the symbol size. Coloured fields represent different HDF compositions, typically analysed using offline LA-ICPMS (cf. legend)[4–8]. Grey diamonds show invisible-fluid compositions in gem-quality diamonds[9]. Blue crosses represent HDFs in fibrous diamond analysed with an online LA-ICPMS approach[10]. Black lines indicate primitive mantle ratios.*

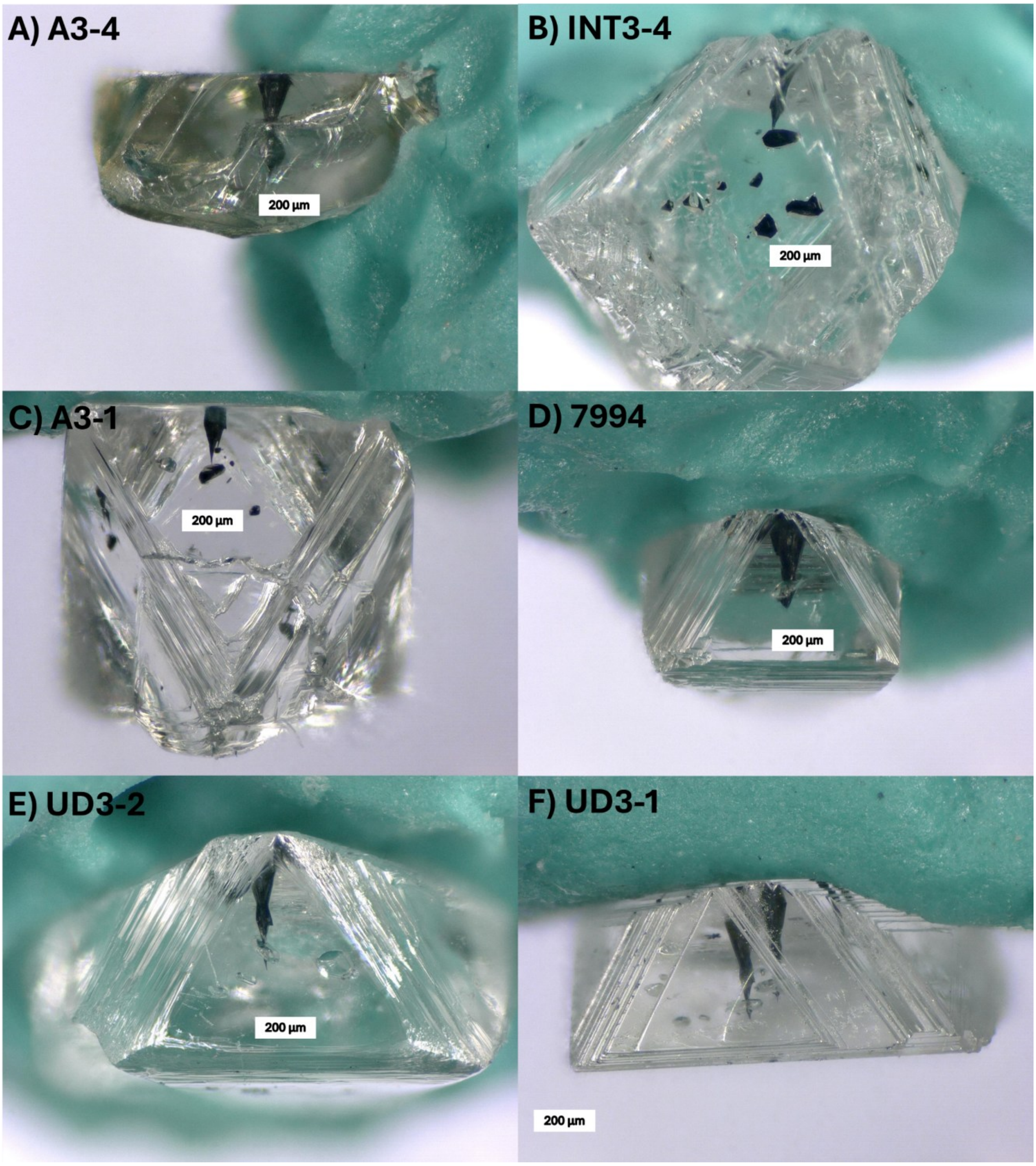

Supplementary Figure 10 Laser ablation pit images of diamond samples. *Post laser ablation (LA) side-view images of diamond samples showing the ablation pits. Images (A) and (D-F) illustrate successfully targeted measurements, where the LA reached the inclusions, whereas (B) and (C) show pits that thinned out before the inclusions were reached by ablation.*

References

1. Nimis, P. *et al.* First evidence of hydrous silicic fluid films around solid inclusions in gem-quality diamonds. *Lithos* **260**, (2016).

2. Sun, Q. The Raman OH stretching bands of liquid water. *Vib. Spectrosc.* **51**, 213–217 (2009).

3. Besemer, M., Bloemenkamp, R., Ariese, F. & Manen, H.-J. van. Identification of Multiple Water–Iodide Species in Concentrated NaI Solutions Based on the Raman Bending Vibration of Water. *J. Phys. Chem. A* **120**, 709–714 (2016).

4. Klein-BenDavid, O. *et al.* Mixed fluid sources involved in diamond growth constrained by Sr–Nd–Pb–C–N isotopes and trace elements. *Earth Planet. Sci. Lett.* **289**, 123–133 (2010).

5. Weiss, Y., Griffin, W. L. & Navon, O. Diamond-forming fluids in fibrous diamonds: The trace-element perspective. *Earth Planet. Sci. Lett.* **376**, 110–125 (2013).

6. Klein-BenDavid, O. *et al.* The sources and time-integrated evolution of diamond-forming fluids – Trace elements and isotopic evidence. *Geochim. Cosmochim. Acta* **125**, 146–169 (2014).

7. Weiss, Y., McNeill, J., Pearson, D. G., Nowell, G. M. & Ottley, C. J. Highly saline fluids from a subducting slab as the source for fluid-rich diamonds. *Nature* **524**, 339–342 (2015).

8. Smith, E. M., Kopylova, M. G., Nowell, G. M., Pearson, D. G. & Ryder, J. Archean mantle fluids preserved in fibrous diamonds from Wawa, Superior craton. *Geology* **40**, 1071–1074 (2012).

9. Krebs, M. Y. *et al.* A common parentage-low abundance trace element data of *gem* diamonds reveals similar fluids to fibrous diamonds. *Lithos* **324–325**, 356–370 (2019).

10. Tomlinson, E. L., Müller, W., & Eimf. A snapshot of mantle metasomatism: Trace element analysis of coexisting fluid (LA-ICP-MS) and silicate (SIMS) inclusions in fibrous diamonds. *Earth Planet. Sci. Lett.* **279**, 362–372 (2009).
